# Supplementary material for: Caenorhabditis elegans processes sensory information to choose between freeloading and self-defense strategies
Source: eLife. 2020 May 5;9:e56186. doi: 10.7554/eLife.56186 (PMC7213980; doi:10.7554/eLife.56186)
Supplement: Supplementary file 1. [file elife-56186-supp1.docx]

| **Supplementary file 1. Statistical analysis for Figure 1 and Figure 1—figure supplement 1.** | | | | | |  |  |  |
| --- | --- | --- | --- | --- | --- | --- | --- | --- |
|  |  |  |  |  |  |  |  |  |
| **Set** | **Genotype** | **Mean survival ± SEM (days)** | **Median survival (days)** | **75th percentile (days)** | **N dead  / initial N** | **% Mean survival change  vs. control** | ***P* value (log-rank) vs. control** | **Figure** |
| Cilium structure | | | | | | | | |
|  | *osm-5(p813) X* | 1.06 ± 0.02 | 1.05 | 1.21 | 123 / 123 | 45% | < 0.0001 | 1 |
|  | wild type | 0.73 ± 0.02 | 0.70 | 0.83 | 118 / 118 |  |  |  |
| Sensory transduction | | | | | | | | |
|  | *tax-4(p678) III* | 1.61 ± 0.03 | 1.64 | 1.79 | 138 / 138 | 124% | < 0.0001 | 1 |
|  | wild type | 0.72 ± 0.02 | 0.68 | 0.83 | 111 / 111 |  |  |  |
|  | *tax-2(p671) I* | 1.01 ± 0.02 | 0.96 | 1.16 | 110 / 110 | 33% | < 0.0001 | S1A |
|  | wild type | 0.76 ± 0.02 | 0.73 | 0.88 | 113 / 113 |  |  |  |
| Neuronal genetic ablation | | | | | | | | |
|  | ASG: ablation | 1.19 ± 0.02 | 1.21 | 1.32 | 138 / 149 | 48% | < 0.0001 | 1 |
|  | wild type | 0.80 ± 0.02 | 0.75 | 0.90 | 127 / 143 |  |  |  |
|  | ASE: *che-1(ot75) I* | 0.91 ± 0.01 | 0.91 | 1.00 | 138 / 138 | 2% | > 0.05 | 1 |
|  | wild type | 0.89 ± 0.01 | 0.89 | 0.98 | 144 / 144 |  |  |  |
|  | ASI: ablation | 1.41 ± 0.02 | 1.44 | 1.50 | 117 / 128 | 61% | < 0.0001 | 1 |
|  | wild type | 0.88 ± 0.01 | 0.86 | 0.98 | 130 / 138 |  |  |  |
|  | ASK: ablation | 0.99 ± 0.02 | 0.96 | 1.20 | 129 / 141 | 13% | < 0.0001 | 1 |
|  | wild type | 0.88 ± 0.02 | 0.87 | 1.00 | 134 / 144 |  |  |  |
|  | IL2: ablation | 1.46 ± 0.03 | 1.39 | 1.71 | 131 / 131 | 39% | < 0.0001 | 1 |
|  | wild type | 1.06 ± 0.02 | 1.05 | 1.17 | 131 / 131 |  |  |  |
|  | ASJ: ablation | 1.16 ± 0.03 | 1.10 | 1.33 | 82 / 82 | -14% | 0.0016 | 1 |
|  | unablated (no transgene) | 1.34 ± 0.04 | 1.35 | 1.63 | 93 / 93 |  |  |  |
|  | AFD: ablation | 1.29 ± 0.03 | 1.27 | 1.47 | 138 / 138 | 39% | < 0.0001 | 1 |
|  | wild type | 0.92 ± 0.02 | 0.89 | 1.06 | 137 / 137 |  |  |  |
|  | AFD: ablation (transgene) | 1.39 ± 0.04 | 1.35 | 1.65 | 75 / 75 | 32% | < 0.0001 | S1B |
|  | unablated (no transgene) | 1.05 ± 0.02 | 1.04 | 1.18 | 147 / 147 |  |  |  |
|  | AWA: ablation | 0.71 ± 0.02 | 0.71 | 0.83 | 72 / 72 | -16% | < 0.0001 | 1 |
|  | unablated (no transgene) | 0.84 ± 0.02 | 0.86 | 0.95 | 80 / 80 |  |  |  |
|  | AWB: ablation | 0.78 ± 0.02 | 0.77 | 0.91 | 106 / 106 | -4% | > 0.05 | 1 |
|  | wild type | 0.82 ± 0.02 | 0.81 | 0.94 | 114 / 114 |  |  |  |
|  | AWC: ablation | 0.89 ± 0.02 | 0.88 | 1.02 | 137 / 137 | 8% | 0.0037 | 1 |
|  | wild type | 0.83 ± 0.01 | 0.83 | 0.92 | 139 / 139 |  |  |  |
|  | ASH: ablation | 0.89 ± 0.02 | 0.89 | 1.01 | 87 / 87 | 4% | > 0.05 | 1 |
|  | wild type | 0.86 ± 0.02 | 0.83 | 0.95 | 128 / 128 |  |  |  |
|  | ADE/PDE/CEP: ablation | 0.85 ± 0.03 | 0.83 | 0.99 | 101 / 101 | 13% | 0.0065 | S1C |
|  | wild type | 0.75 ± 0.02 | 0.71 | 0.88 | 140 / 140 |  |  |  |
|  | ADL: ablation | 1.83 ± 0.04 | 1.83 | 2.01 | 86 / 86 | -6% | > 0.05 | S1D |
|  | wild type | 1.94 ± 0.04 | 1.88 | 2.13 | 97 / 97 |  |  |  |
|  | ADF: ablation | 0.88 ± 0.02 | 0.86 | 0.98 | 55 / 55 | 2% | > 0.05 | S1E |
|  | wild type | 0.86 ± 0.02 | 0.82 | 0.94 | 56 / 56 |  |  |  |
|  | OLL: ablation | 1.04 ± 0.03 | 1.00 | 1.14 | 56 / 56 | -1% | > 0.05 | S1F |
|  | unablated (no transgene) | 1.05 ± 0.02 | 1.04 | 1.15 | 132 / 132 |  |  |  |
|  | ALM/PLM/AVM/PVM/FLP/PVD: ablation (transgene) | 1.17 ± 0.05 | 1.16 | 1.45 | 68 / 68 | -6% | > 0.05 | S1G |
|  | unablated (no transgene) | 1.24 ± 0.02 | 1.25 | 1.36 | 115 / 115 |  |  |  |
|  | URX/AQR/PQR: ablation | 0.83 ± 0.02 | 0.79 | 0.98 | 145 / 145 | -15% | < 0.0001 | 1 |
|  | wild type | 0.98 ± 0.02 | 0.97 | 1.12 | 142 / 142 |  |  |  |
