# Supplementary material for: Caenorhabditis elegans processes sensory information to choose between freeloading and self-defense strategies
Source: eLife. 2020 May 5;9:e56186. doi: 10.7554/eLife.56186 (PMC7213980; doi:10.7554/eLife.56186)
Supplement: Supplementary file 2. [file elife-56186-supp2.docx]

| **Supplementary file 2. Statistical analysis for Figure 2 and Figure 2—figure supplement 1.** | | | | | |  |  |  |  |  |  |
| --- | --- | --- | --- | --- | --- | --- | --- | --- | --- | --- | --- |
|  |  |  |  |  |  |  |  |  |  |  |  |
| **Set** | **Genotype** | **Mean survival ± SEM (days)** | **Median survival (days)** | **75th percentile (days)** | **N dead  / initial N** | **% Mean survival change  vs.  wild type** | **Group** | ***P* value  (log-rank) vs.  wild type** | ***P* value (log-rank) vs.  group b** | ***P* value (log-rank) vs.  group c** | **Figure** |
| 6 mM tBuOOH, 25°C, OP50 | | | | | | | | | | | |
|  | wild type | 0.92 ± 0.02 | 0.90 | 1.02 | 98 / 98 |  | a |  |  |  | 2B |
|  | *daf-7(ok3125) III* (no *qdEx37* transgene) | 1.88 ± 0.04 | 1.94 | 2.10 | 83 / 83 | 104% | b | < 0.0001 |  |  |  |
|  | *daf-7(ok3125) III; qdEx37[Pdaf-7::daf-7(+), Pges-1::GFP]* | 0.97 ± 0.02 | 0.95 | 1.06 | 117 / 117 | 5% | c | > 0.05 | < 0.0001 | < 0.0001 |  |
|  | wild type | 0.97 ± 0.02 | 0.94 | 1.10 | 109 / 109 |  | a |  |  |  | 2C,D |
|  | *daf-7(ok3125) III* (no q*dEx44* transgene) | 2.35 ± 0.05 | 2.41 | 2.66 | 89 / 89 | 142% | b | < 0.0001 |  |  |  |
|  | *daf-7(ok3125) III; qdEx44[Pstr-3::daf-7(+), Pges-1::GFP]* | 1.05 ± 0.03 | 1.05 | 1.17 | 80 / 80 | 9% | c | 0.0031 | < 0.0001 | < 0.0001 |  |
|  | *daf-7(ok3125) III* (no q*dEx40* transgene) | 2.21 ± 0.07 | 2.32 | 2.50 | 44 / 44 | 128% | d | < 0.0001 |  |  |  |
|  | *daf-7(ok3125) III; qdEx40[Ptrx-1::daf-7(+), Pges-1::GFP]* | 0.99 ± 0.02 | 0.98 | 1.11 | 70 / 70 | 2% | e | > 0.05 | < 0.0001 | < 0.0001 |  |
|  | wild type | 0.84 ± 0.02 | 0.86 | 0.92 | 78 / 81 |  | a |  |  |  | S2A |
|  | *daf-7(e1372) III* | 1.99 ± 0.04 | 1.99 | 2.29 | 94 / 101 | 137% | b | < 0.0001 |  |  |  |
| 6 mM tBuOOH, 20°C, OP50 | | | | | | | | | | | |
|  | wild type | 1.71 ± 0.06 | 1.73 | 1.98 | 67 / 68 |  | a |  |  |  | S2B |
|  | *daf-7(e1372) III* | 4.81 ± 0.17 | 4.95 | 5.90 | 73 / 79 | 181% | b | < 0.0001 |  |  |  |
| 6 mM tBuOOH, 25°C, HT115 | | | | | | | | | | | |
|  | wild type | 0.82 ± 0.02 | 0.81 | 0.94 | 121 / 121 |  | a |  |  |  | S2C |
|  | *daf-7(e1372) III* | 1.87 ± 0.05 | 1.84 | 2.17 | 111 / 111 | 127% | b | < 0.0001 |  |  |  |
| 6 mM tBuOOH, 25°C, HT115 *egg-5(RNAi)*, no FUDR | | | | | | | | | | | |
|  | wild type | 0.86 ± 0.04 | 0.85 | 1.13 | 55 / 61 |  | a |  |  |  | S2D |
|  | *daf-7(ok3125) III* | 1.85 ± 0.08 | 1.87 | 2.23 | 48 / 53 | 113% | b | < 0.0001 |  |  |  |
|  | *daf-1(m40) IV* | 2.25 ± 0.13 | 2.31 | 3.00 | 46 / 52 | 160% | c | < 0.0001 |  |  |  |
| 5 mM arsenite, 25°C, OP50 | | | | | | | | | | | |
|  | wild type | 1.27 ± 0.02 | 1.26 | 1.37 | 117 / 117 |  | a |  |  |  | 2E |
|  | *daf-7(ok3125) III* | 1.22 ± 0.03 | 1.23 | 1.42 | 111 / 111 | -4% | b | > 0.05 |  |  |  |
| 75 mM paraquat, 25°C, OP50 | | | | | | | | | | | |
|  | wild type | 1.97 ± 0.05 | 2.00 | 2.37 | 90 / 90 |  | a |  |  |  | 2F |
|  | *daf-7(ok3125) III* | 2.11 ± 0.07 | 2.14 | 2.57 | 93 / 93 | 7% | b | > 0.05 |  |  |  |
| 25 mM dithiothreitol (DTT), 25°C, OP50 | | | | | | | | | | | |
|  | wild type | 0.37 ± 0.01 | 0.36 | 0.42 | 111 / 116 |  | a |  |  |  | 2G |
|  | *daf-7(ok3125) III* | 0.40 ± 0.01 | 0.39 | 0.48 | 110 / 110 | 7% | b | > 0.05 |  |  |  |
