# Supplementary material for: Caenorhabditis elegans processes sensory information to choose between freeloading and self-defense strategies
Source: eLife. 2020 May 5;9:e56186. doi: 10.7554/eLife.56186 (PMC7213980; doi:10.7554/eLife.56186)
Supplement: Supplementary file 3. [file elife-56186-supp3.docx]

| **Supplementary file 3 . Statistical analysis for Figure 3 and Figure 3—figure supplement 1.** | | | | | |  |  |  |  |  |  |
| --- | --- | --- | --- | --- | --- | --- | --- | --- | --- | --- | --- |
|  |  |  |  |  |  |  |  |  |  |  |  |
| **Set** | **Genotype** | **Mean survival ± SEM (days)** | **Median survival (days)** | **75th percentile (days)** | **N dead  / initial N** | **Group** | **% Mean survival change  vs.  group a** | ***P* value  (log-rank) vs.  group a** | ***P* value (log-rank) vs.  group b** | ***P* value (log-rank) vs.  group c** | **Figure** |
| Pathway analysis | | | | | | | | | | | |
|  | wild type | 0.88 ± 0.02 | 0.82 | 0.99 | 122 / 122 | a |  |  |  |  | 3A |
|  | *daf-1(m40) IV* | 2.69 ± 0.03 | 2.69 | 2.84 | 102 / 102 | b | 207% | < 0.0001 |  |  |  |
|  | *daf-3(mgDf90) X* | 0.91 ± 0.02 | 0.90 | 1.04 | 130 / 130 | c | 3% | > 0.05 | < 0.0001 |  |  |
|  | *daf-1(m40) IV; daf-3(mgDf90) X* | 1.04 ± 0.02 | 1.04 | 1.15 | 127 / 127 | d | 19% | < 0.0001 | < 0.0001 | < 0.0001 |  |
|  | wild type | 0.66 ± 0.01 | 0.66 | 0.73 | 156 / 163 | a |  |  |  |  | S3A |
|  | *daf-7(ok3125) III* | 2.15 ± 0.03 | 2.21 | 2.49 | 154 / 176 | b | 223% | < 0.0001 |  |  |  |
|  | *daf-3(e1376) X* | 0.57 ± 0.01 | 0.55 | 0.64 | 139 / 143 | c | -14% | < 0.0001 | < 0.0001 |  |  |
|  | *daf-7(ok3125) III; daf-3(e1376) X* | 0.73 ± 0.01 | 0.73 | 0.84 | 151 / 157 | d | 10% | < 0.0001 | < 0.0001 | < 0.0001 |  |
|  | wild type | 1.11 ± 0.02 | 1.07 | 1.25 | 121 / 121 | a |  |  |  |  | S3B |
|  | *daf-7(e1372) III* | 3.63 ± 0.08 | 3.65 | 4.17 | 107 / 107 | b | 226% | < 0.0001 |  |  |  |
|  | *daf-5(e1386) II* | 1.34 ± 0.03 | 1.35 | 1.51 | 72 / 72 | c | 21% | < 0.0001 | < 0.0001 |  |  |
|  | *daf-5(e1386) II; daf-7(e1372) III* | 1.39 ± 0.02 | 1.38 | 1.54 | 128 / 128 | d | 25% | < 0.0001 | < 0.0001 | > 0.05 |  |
|  | wild type | 0.93 ± 0.02 | 0.91 | 1.08 | 137 / 137 | a |  |  |  |  | S3C |
|  | *daf-1(m40) IV* | 2.32 ± 0.07 | 2.20 | 2.77 | 98 / 98 | b | 149% | < 0.0001 |  |  |  |
|  | *daf-5(e1386) II* | 1.20 ± 0.02 | 1.41 | 1.59 | 106 / 106 | c | 29% | < 0.0001 | < 0.0001 |  |  |
|  | *daf-5(e1386) II; daf-1(m40) IV* | 1.40 ± 0.02 | 1.19 | 1.38 | 105 / 105 | d | 50% | < 0.0001 | < 0.0001 | < 0.0001 |  |
|  | wild type | 0.99 ± 0.01 | 0.99 | 1.10 | 161 / 161 | a |  |  |  |  | 3B |
|  | ASI ablation | 1.52 ± 0.03 | 1.50 | 1.71 | 120 / 120 | b | 54% | < 0.0001 |  |  |  |
|  | *daf-3(mgDf90) X* | 0.93 ± 0.02 | 0.93 | 1.07 | 150 / 150 | c | -6% | 0.03 | < 0.0001 |  |  |
|  | ASI ablation*; daf-3(mgDf90) X* | 1.00 ± 0.02 | 1.02 | 1.20 | 156 / 156 | d | 2% | 0.0045 | < 0.0001 | < 0.0001 |  |
| Full DAF-1 expression | | | | | | | | | | | |
|  | *daf-1(m40) IV* (no *ftEx98* transgene) | 2.58 ± 0.06 | 2.76 | 3.00 | 92 / 94 | a |  |  |  |  | 3D |
|  | *daf-1(m40) IV; ftEx98[Pdaf-1::daf-1-gfp Podr-1::dsRED]* | 1.54 ± 0.03 | 1.51 | 1.76 | 115 / 121 | b | -40% | < 0.0001 |  |  |  |
| DAF-1 in neurons | | | | | | | | | | | |
|  | *daf-1(m40) IV (no ftEx69 transgene)* | 3.00 ± 0.05 | 3.03 | 3.29 | 91 / 91 | a |  |  |  |  | 3E |
|  | *daf-1(m40); ftEx69[Pegl-3::daf-1::GFP + Podr-1::dsRED]* | 2.13 ± 0.04 | 2.06 | 2.38 | 109 / 109 | b | -29% | < 0.0001 |  |  |  |
| DAF-1 in ciliated neurons | | | | | | | | | | | |
|  | *daf-1(m40) IV* (no *ftEx83* transgene) | 2.41 ± 0.06 | 2.51 | 2.89 | 114 / 126 | a |  |  |  |  | 3F |
|  | *daf-1(m40ts) IV; ftEx83[Posm-6::daf-1-gfp + Podr-1::dsRED]* | 2.27 ± 0.05 | 2.30 | 2.69 | 128 / 133 | b | -6% | 0.0048 |  |  |  |
| DAF-1 in AVK (set 1) | | | | | | | | | | | |
|  | *daf-1(m40) IV* (no *ftEx166* transgene) | 2.58 ± 0.08 | 2.43 | 3.33 | 114 / 114 | a |  |  |  |  | 3G |
|  | *daf-1(m40) IV; ftEx166[Pflp-1::daf-1::gfp + Podr-1::dsRED]* | 1.80 ± 0.04 | 1.73 | 1.95 | 83 / 83 | b | -30% | < 0.0001 |  |  |  |
| DAF-1 in set 2 | | | | | | | | | | | |
|  | *daf-1(m40) IV (no ftEx205 transgene)* | 2.83 ± 0.09 | 2.85 | 3.20 | 61 / 61 | a |  |  |  |  | 3H |
|  | *daf-1(m40) IV; ftEx205[Ptdc-1::daf-1-gfp + Podr-1::dsRed]* | 1.68 ± 0.04 | 1.71 | 1.86 | 97 / 97 | b | -41% | < 0.0001 |  |  |  |
| DAF-1 in set 3 | | | | | | | | | | | |
|  | *daf-1(m40) IV* (no *ftEx93* transgene) | 2.77 ± 0.06 | 2.88 | 3.31 | 136 / 136 | a |  |  |  |  | 3I |
|  | *daf-1(m40) IV; ftEx93[Pglr-1::daf-1-gfp + Podr-1::dsRED]* | 1.84 ± 0.03 | 1.83 | 2.05 | 131 / 131 | b | -34% | < 0.0001 |  |  |  |
| DAF-1 in set 4 | | | | | | | | | | | |
|  | *daf-1(m40) IV* (no *ftEx74* transgene) | 2.47 ± 0.04 | 2.54 | 2.73 | 80 / 80 | a |  |  |  |  | S3D |
|  | *daf-1(m40); ftEx74[Pglr-8p::daf-1-GFP + Podr-1::dsRED]* | 1.86 ± 0.03 | 1.84 | 2.04 | 103 / 103 | b | -25% | < 0.0001 |  |  |  |
| DAF-1 in set 5 | | | | | | | | | | | |
|  | *daf-1(m40) IV* (no *ftEx183* transgene) | 2.72 ± 0.06 | 2.75 | 3.19 | 99 / 99 | a |  |  |  |  | S3E |
|  | *daf-1(m40) IV; ftEx183[Pglr-7::daf-1-gfp + Podr-1::dsRED]* | 2.68 ± 0.06 | 2.64 | 3.20 | 105 / 105 | b | -1% | > 0.05 |  |  |  |
| DAF-3 in DAF-1 cells | | | | | | | | | | | |
|  | *daf-1(m40) IV; daf-3(mgDf90) X* | 0.89 ± 0.04 | 0.93 | 1.02 | 35 / 35 | a |  |  |  |  | 3J |
|  | *daf-1(m40) IV; daf-3(mgDf90) X; witEx4[Pdaf-1::daf-3 + Punc-122::RFP]* | 1.27 ± 0.04 | 1.31 | 1.42 | 47 / 47 | b | 43% | < 0.0001 |  |  |  |
| DAF-3 in set 2 | | | | | | | | | | | |
|  | *daf-1(m40) IV; daf-3(mgDf90) X* | 0.96 ± 0.03 | 0.89 | 1.03 | 42 / 42 | a |  |  |  |  | 3K |
|  | *daf-1(m40) IV; daf-3(mgDf90) X; witEx2[Ptdc-1::daf-3 + Pmyo-2::RFP]* | 0.88 ± 0.04 | 0.85 | 1.09 | 54 / 54 | b | -8% | > 0.05 |  |  |  |
|  | *daf-1(m40) IV; daf-3(mgDf90) X* | 0.80 ± 0.02 | 0.78 | 0.89 | 38 / 40 | a |  |  |  |  | S3F |
|  | *daf-1(m40) IV; daf-3(mgDf90) X; witEx3[Ptdc-1::daf-3 + Pmyo-2::RFP]* line 1 | 0.75 ± 0.04 | 0.67 | 0.86 | 25 / 26 | b | -4% | > 0.05 |  |  |  |
|  | *daf-1(m40) IV; daf-3(mgDf90) X; witEx2[Ptdc-1::daf-3 + Pmyo-2::RFP]* line 2 | 0.76 ± 0.03 | 0.72 | 0.85 | 38 / 40 | c | -4% | > 0.05 |  |  |  |
|  | *daf-1(m40) IV; daf-3(mgDf90) X; witEx1[Ptdc-1::daf-3 + Pmyo-2::RFP]* line 3 | 0.66 ± 0.04 | 0.68 | 0.76 | 26 / 26 | d | -17% | 0.02 |  |  |  |
