# Supplementary material for: Caenorhabditis elegans processes sensory information to choose between freeloading and self-defense strategies
Source: eLife. 2020 May 5;9:e56186. doi: 10.7554/eLife.56186 (PMC7213980; doi:10.7554/eLife.56186)
Supplement: Supplementary file 4. [file elife-56186-supp4.docx]

| **Supplementary file 4. Statistical analysis for Figure 4 and Figure 4—figure supplement 1.** | | | | | |  |  |  |  |  |  |  |
| --- | --- | --- | --- | --- | --- | --- | --- | --- | --- | --- | --- | --- |
|  |  |  |  |  |  |  |  |  |  |  |  |  |
| **Set** | **Genotype** | **Mean survival ± SEM (days)** | **Median survival (days)** | **75th percentile (days)** | **N dead  / initial N** | **Group** | **% Mean survival change  vs.  group a** | ***P* value  (log-rank) vs.  group a** | ***P* value (log-rank) vs.  group b** | ***P* value (log-rank) vs.  group c** | ***P* value (log-rank) vs.  group e** | **Figure** |
| Dauer | | | | | | | | | | | | |
|  | wild type | 0.94 ± 0.02 | 0.91 | 1.09 | 118 / 118 | a |  |  |  |  |  | 4B |
|  | *daf-7(e1372) III* | 2.05 ± 0.07 | 1.91 | 2.41 | 81 / 81 | b | 118% | < 0.0001 |  |  |  |  |
|  | *daf-12(rh61rh411) X* | 0.82 ± 0.02 | 0.78 | 0.91 | 59 / 59 | c | -13% | < 0.0001 | < 0.0001 |  |  |  |
|  | *daf-7(e1372) III; daf-12(rh61rh411) X* | 3.02 ± 0.09 | 3.04 | 3.64 | 82 / 82 | d | 222% | < 0.0001 | < 0.0001 | < 0.0001 |  |  |
| Fat storage | | | | | | | | | | | | |
|  | wild type | 0.95 ± 0.01 | 0.92 | 1.08 | 235 / 235 | a |  |  |  |  |  | 4C |
|  | *daf-1(m40) IV* | 2.45 ± 0.05 | 2.34 | 2.87 | 193 / 200 | b | 158% | < 0.0001 |  |  |  |  |
|  | *mgl-3(tm1766) IV; mgl-1(tm1811) X* | 0.98 ± 0.02 | 0.93 | 1.06 | 125 / 125 | c | 3% | > 0.05 | < 0.0001 |  |  |  |
|  | *daf-1(m40) mgl-3(tm1766) IV; mgl-1(tm1811) X* | 2.36 ± 0.04 | 2.36 | 2.79 | 226 / 226 | d | 148% | < 0.0001 | > 0.05 | < 0.0001 |  |  |
|  | wild type | 0.96 ± 0.01 | 0.95 | 1.08 | 340 / 340 | a |  |  |  |  |  | S4A,B |
|  | *daf-1(m40) IV* | 2.44 ± 0.04 | 2.40 | 2.87 | 291 / 298 | b | 155% | < 0.0001 |  |  |  |  |
|  | *mgl-1(tm1811) X* | 0.85 ± 0.02 | 0.84 | 0.93 | 101 / 101 | c | -11% | < 0.0001 | < 0.0001 |  |  |  |
|  | *mgl-1(tm1811) X; daf-1(m40) IV* | 2.33 ± 0.04 | 2.37 | 2.60 | 94 / 94 | d | 143% | < 0.0001 | 0.001 | < 0.0001 |  |  |
|  | *mgl-3(tm1766) IV* | 1.17 ± 0.02 | 1.17 | 1.33 | 98 / 103 | e | 22% | < 0.0001 | < 0.0001 |  |  |  |
|  | *daf-1(m40) mgl-3(tm1766) IV* | 2.39 ± 0.06 | 2.35 | 2.86 | 100 / 100 | f | 149% | < 0.0001 | > 0.05 |  | < 0.0001 |  |
| Germline | | | | | | | | | | | | |
|  | *mes-1(ok2467) X* (fertile) | 0.97 ± 0.03 | 0.92 | 1.10 | 97 / 97 | a |  |  |  |  |  | 4D |
|  | *daf-1(m40) IV; mes-1(ok2467) X* (fertile) | 2.39 ± 0.05 | 2.44 | 2.67 | 85 / 85 | b | 147% | < 0.0001 |  |  |  |  |
|  | *mes-1(ok2467) X* (sterile) | 1.52 ± 0.04 | 1.55 | 1.80 | 105 / 105 | c | 57% | < 0.0001 | < 0.0001 |  |  |  |
|  | *daf-1(m40) IV; mes-1(ok2467) X* (sterile) | 2.89 ± 0.12 | 2.94 | 3.57 | 52 / 52 | d | 198% | < 0.0001 | < 0.0001 | < 0.0001 |  |  |
|  | *mes-1(ok2467) X* (fertile) | 0.93 ± 0.02 | 0.91 | 1.05 | 123 / 123 | a |  |  |  |  |  | 4E |
|  | *daf-3(mgDf90) mes-1(ok2467) X* (fertile) | 0.96 ± 0.02 | 0.96 | 1.09 | 126 / 126 | b | 3% | > 0.05 |  |  |  |  |
|  | *mes-1(ok2467) X* (sterile) | 1.44 ± 0.03 | 1.49 | 1.67 | 126 / 126 | c | 54% | < 0.0001 | < 0.0001 |  |  |  |
|  | *daf-3(mgDf90) mes-1(ok2467) X* (sterile) | 1.36 ± 0.03 | 1.36 | 1.53 | 123 / 123 | d | 45% | < 0.0001 | < 0.0001 | > 0.05 |  |  |
