# Supplementary material for: Caenorhabditis elegans processes sensory information to choose between freeloading and self-defense strategies
Source: eLife. 2020 May 5;9:e56186. doi: 10.7554/eLife.56186 (PMC7213980; doi:10.7554/eLife.56186)
Supplement: Supplementary file 5. [file elife-56186-supp5.docx]

| **Supplementary file 5. Statistical analysis for Figure 5 and Figure 5—figure supplement 1.** | | | | | | | |  |  |  |  |
| --- | --- | --- | --- | --- | --- | --- | --- | --- | --- | --- | --- |
|  |  |  |  |  |  |  |  |  |  |  |  |
| **Set** | **Genotype** | **Mean survival ± SEM (days)** | **Median survival (days)** | **75th percentile (days)** | **N dead  / initial N** | **Group** | **% Mean survival change  vs.  group a** | ***P* value  (log-rank) vs.  group a** | ***P* value (log-rank) vs.  group b** | ***P* value (log-rank) vs.  group c** | **Figure** |
| Pathway analysis | | | | | | | | | | | |
|  | wild type | 0.89 ± 0.01 | 0.87 | 1.03 | 275 / 275 | a |  |  |  |  | 5A |
|  | *ins-4(tm3620)* | 1.16 ± 0.02 | 1.18 | 1.32 | 120 / 120 | b | 30% | < 0.0001 |  |  |  |
|  | *ins-5(tm2560) II* | 1.09 ± 0.03 | 1.11 | 1.28 | 86 / 86 | c | 22% | < 0.0001 |  |  |  |
|  | *ins-6(tm2416) II* | 1.47 ± 0.03 | 1.49 | 1.69 | 90 / 90 | d | 65% | < 0.0001 |  |  |  |
|  | *daf-28(tm2308) V* | 1.18 ± 0.02 | 1.15 | 1.30 | 137 / 137 | e | 32% | < 0.0001 |  |  |  |
|  | wild type | 0.77 ± 0.01 | 0.74 | 0.83 | 128 / 128 | a |  |  |  |  | S5A |
|  | *ins-1(nr2091) IV* | 0.95 ± 0.02 | 0.94 | 1.04 | 113 / 113 | b | 24% | < 0.0001 |  |  |  |
|  | wild type | 0.98 ± 0.02 | 0.96 | 1.10 | 147 / 147 | a |  |  |  |  | S5B |
|  | *ins-3(tm3608) II* | 1.08 ± 0.02 | 1.10 | 1.23 | 141 / 141 | b | 11% | < 0.0001 |  |  |  |
|  | wild type | 1.03 ± 0.01 | 1.03 | 1.12 | 129 / 140 | a |  |  |  |  | 5B |
|  | *daf-1(m40) IV* | 2.79 ± 0.08 | 2.76 | 3.63 | 160 / 183 | b | 171% | < 0.0001 |  |  |  |
|  | *daf-2(e1370) III* | 3.02 ± 0.06 | 2.87 | 3.57 | 166 / 185 | c | 193% | < 0.0001 | > 0.05 |  |  |
|  | *daf-2(e1370) III; daf-1(m40) IV* | 5.88 ± 0.21 | 5.63 | 7.12 | 77 / 159 | d | 471% | < 0.0001 | < 0.0001 | < 0.0001 |  |
|  | wild type | 0.84 ± 0.01 | 0.82 | 0.92 | 145 / 158 | a |  |  |  |  | 5C |
|  | *daf-2(e1370) III* | 3.50 ± 0.06 | 3.46 | 4.07 | 136 / 154 | b | 318% | < 0.0001 |  |  |  |
|  | *daf-16(mu86) I* | 0.78 ± 0.01 | 0.76 | 0.86 | 141 / 153 | c | -7% | 0.004 | < 0.0001 |  |  |
|  | *daf-16(mu86) I; daf-2(e1370) III* | 0.72 ± 0.01 | 0.71 | 0.79 | 140 / 151 | d | -14% | < 0.0001 | < 0.0001 | < 0.0001 |  |
|  | *ayIs7[Phlh-8::GFP + dpy-20(+)]* | 0.83 ± 0.01 | 0.84 | 0.92 | 145 / 145 | a |  |  |  |  | 5D |
|  | *ins-4 ins-5 ins-6(hpDf761) II; ayIs7 IV; daf-28(tm2308) V* | 1.26 ± 0.02 | 1.25 | 1.45 | 131 / 131 | b | 52% | < 0.0001 |  |  |  |
|  | *daf-16(mgDf50) I* | 0.77 ± 0.01 | 0.73 | 0.83 | 133 / 133 | c | -8% | 0.012 | < 0.0001 |  |  |
|  | *daf-16(mgDf50) I; ins-4 ins-5 ins-6(hpDf761) II; ayIs7 IV; daf-28(tm2308) V* | 0.69 ± 0.01 | 0.69 | 0.78 | 140 / 140 | d | -17% | < 0.0001 | < 0.0001 | < 0.0001 |  |
|  | wild type | 0.89 ± 0.02 | 0.86 | 0.98 | 159 / 161 | a |  |  |  |  | 5E |
|  | *daf-7(e1372) III* | 2.46 ± 0.06 | 2.44 | 2.91 | 114 / 118 | b | 175% | < 0.0001 |  |  |  |
|  | *daf-16(mu86) I* | 0.68 ± 0.01 | 0.67 | 0.73 | 163 / 165 | c | -24% | < 0.0001 | < 0.0001 |  |  |
|  | *daf-16(mu86) I; daf-7(e1372) III* | 1.16 ± 0.02 | 1.15 | 1.30 | 160 / 168 | d | 29% | < 0.0001 | < 0.0001 | < 0.0001 |  |
|  | wild type | 0.99 ± 0.01 | 0.98 | 1.09 | 143 / 143 | a |  |  |  |  | 5F |
|  | *daf-1(m40) IV* | 2.45 ± 0.05 | 2.57 | 2.79 | 127 / 127 | b | 147% | < 0.0001 |  |  |  |
|  | *daf-16(mu86) I* | 0.84 ± 0.01 | 0.83 | 0.93 | 105 / 105 | c | -15% | < 0.0001 | < 0.0001 |  |  |
|  | *daf-16(mu86) I; daf-1(m40) IV* | 1.39 ± 0.02 | 1.43 | 1.51 | 123 / 123 | d | 40% | < 0.0001 | < 0.0001 | < 0.0001 |  |
|  | *daf-1(m40) IV* | 2.79 ± 0.08 | 2.78 | 3.50 | 98 / 98 | a |  |  |  |  | S5C |
|  | *daf-1(m40) hlh-30(tm1978) IV* | 2.68 ± 0.06 | 2.65 | 3.04 | 121 / 121 | b | -4% | 0.04 |  |  |  |
|  | wild type + empty vector | 0.90 ± 0.02 | 0.85 | 1.01 | 123 / 123 | a |  |  |  |  | 5G |
|  | *daf-1(m40) IV* + empty vector | 2.15 ± 0.06 | 2.17 | 2.46 | 109 / 109 | b | 140% | < 0.0001 |  |  |  |
|  | *skn-1(RNAi)* | 0.91 ± 0.01 | 0.92 | 1.01 | 130 / 130 | c | 2% | > 0.05 | < 0.0001 |  |  |
|  | *daf-1(m40) IV* + *skn-1(RNAi)* | 1.52 ± 0.04 | 1.53 | 1.80 | 107 / 107 | d | 69% | < 0.0001 | < 0.0001 | < 0.0001 |  |
|  | *daf-16(mu86) I* + empty vector | 0.77 ± 0.02 | 0.77 | 0.89 | 119 / 119 | a |  |  |  |  | 5H |
|  | *daf-16(mu86) I; daf-1(m40) IV* + empty vector | 1.26 ± 0.03 | 1.26 | 1.46 | 92 / 92 | b | 64% | < 0.0001 |  |  |  |
|  | *daf-16(mu86) I* + *skn-1(RNAi)* | 0.57 ± 0.01 | 0.56 | 0.64 | 134 / 134 | c | -26% | < 0.0001 | < 0.0001 |  |  |
|  | *daf-16(mu86) I; daf-1(m40) IV* + *skn-1(RNAi)* | 0.79 ± 0.02 | 0.82 | 1.00 | 114 / 114 | d | 3% | > 0.05 | < 0.0001 | < 0.0001 |  |
| Full DAF-16 expression | | | | | | | | | | | |
|  | *daf-16(mu86) I; daf-1(m40) IV* | 1.44 ± 0.02 | 1.48 | 1.59 | 130 / 130 | a |  |  |  |  | 5I |
|  | *daf-16(mu86) I; daf-1(m40) IV; qyIs288[Pdaf-16::GFP::daf-16 + unc-119(+)]* | 2.62 ± 0.07 | 2.66 | 3.20 | 120 / 120 | b | 82% | < 0.0001 |  |  |  |
|  | *daf-1(m40) IV* | 2.38 ± 0.04 | 2.43 | 2.63 | 88 / 88 | c | 65% | < 0.0001 | < 0.0001 |  |  |
| DAF-16 in intestine | | | | | | | | | | | |
|  | *daf-16(mu86) I; daf-1(m40) IV* | 1.65 ± 0.02 | 1.68 | 1.78 | 71 / 74 | a |  |  |  |  | 5J |
|  | *daf-16(mu86) I; daf-1(m40) IV; qyIs292[Pges-1::GFP::daf-16]* | 2.22 ± 0.03 | 2.17 | 2.47 | 93 / 97 | b | 34% | < 0.0001 |  |  |  |
|  | *daf-1(m40) IV* | 2.72 ± 0.06 | 2.74 | 3.14 | 61 / 72 | c | 64% | < 0.0001 | < 0.0001 |  |  |
| DAF-16 in neurons | | | | | | | | | | | |
|  | *daf-16(mu86) I; daf-1(m40) IV* | 1.63 ± 0.02 | 1.63 | 1.80 | 130 / 130 | a |  |  |  |  | 5K |
|  | *daf-16(mu86) I; daf-1(m40) IV; qyIs294[Punc-119::GFP::daf-16]* | 1.76 ± 0.03 | 1.76 | 1.99 | 134 / 134 | b | 8% | 0.0004 |  |  |  |
|  | *daf-1(m40) IV* | 2.35 ± 0.06 | 2.42 | 2.82 | 124 / 124 | c | 44% | < 0.0001 | < 0.0001 |  |  |
| DAF-16 in body muscle | | | | | | | | | | | |
|  | *daf-16(mu86) I; daf-1(m40) IV* | 1.47 ± 0.02 | 1.48 | 1.61 | 113 / 113 | a |  |  |  |  | 5L |
|  | *daf-16(mu86) I; unc-119(ed4) III; daf-1(m40) IV; qyEx264 [Pmyo-3::GFP::daf-16 + unc-119(+)]* | 1.47 ± 0.03 | 1.49 | 1.63 | 114 / 114 | b | 0% | > 0.05 |  |  |  |
|  | *daf-1(m40) IV* | 2.38 ± 0.06 | 2.45 | 2.91 | 114 / 114 | c | 62% | < 0.0001 | < 0.0001 |  |  |
| DAF-16 in hypodermis | | | | | | | | | | | |
|  | *daf-16(mu86) I; daf-1(m40) IV* | 1.52 ± 0.03 | 1.55 | 1.66 | 49 / 49 | a |  |  |  |  | 5M |
|  | *daf-16(mu86) I; daf-1(m40) IV; qyIs290[Pcol-12::GFP::daf-16 + unc-119(+)]* | 1.13 ± 0.09 | 1.25 | 1.50 | 29 / 29 | b | -26% | 0.008 |  |  |  |
|  | *daf-1(m40) IV* | 2.78 ± 0.05 | 2.78 | 3.12 | 82 / 82 | c | 83% | < 0.0001 | < 0.0001 |  |  |
