# Supplementary material for: Caenorhabditis elegans processes sensory information to choose between freeloading and self-defense strategies
Source: eLife. 2020 May 5;9:e56186. doi: 10.7554/eLife.56186 (PMC7213980; doi:10.7554/eLife.56186)
Supplement: Supplementary file 6. [file elife-56186-supp6.docx]

| **Supplementary file 6. Statistical analysis for Figure 6 and Figure 6—figure supplement 1.** | | |  |  |  |
| --- | --- | --- | --- | --- | --- |
|  |  |  |  |  |  |
| **Gene set** | **Gene set reference** | **Number of genes in set with expression** | **Fold change in gene expression log2(*daf-7(ok3125)* / wildtype) Mean ± SEM** | ***P* value vs.  all genes (ANOVA)** | **Figure** |
| All genes |  | 9660 | -0.11 ± 0.02 |  |  |
| Genes upregulated in TGFβ signaling pathway mutants | Shaw et al., 2007 | 1388 | 1.43 ± 0.02 | < 0.0001 | S6B |
| Genes downregulated in TGFβ signaling pathway mutants | Shaw et al., 2007 | 3490 | -1.44 ± 0.02 | < 0.0001 | S6B |
| Genes upregulated by *skn-1(+)* in wildtype | Oliveira et al., 2009 | 28 | 2.01 ± 0.13 | < 0.0001 | 6C |
| Genes upregulated by *daf-16(+)* in wildtype | Kumar et al., 2015 | 32 | 1.31 ± 0.18 | < 0.0001 | 6D |
| Genes upregulated by *skn-1(+)* in *daf-2(-)* | Ewald et al., 2015 | 158 | 1.35 ± 0.08 | < 0.0001 | S6C |
| Genes upregulated by *daf-16(+)* in *daf-2(-)* | Murphy et al., 2003 | 152 | 1.31 ± 0.08 | < 0.0001 | S6D |
