# Supplementary material for: Caenorhabditis elegans processes sensory information to choose between freeloading and self-defense strategies
Source: eLife. 2020 May 5;9:e56186. doi: 10.7554/eLife.56186 (PMC7213980; doi:10.7554/eLife.56186)
Supplement: Supplementary file 7. [file elife-56186-supp7.docx]

| **Supplementary file 7. Statistical analysis for Figure 7 and Figure 7—figure supplement 1.** | | | | | |  |  |  |  |  |  |
| --- | --- | --- | --- | --- | --- | --- | --- | --- | --- | --- | --- |
|  |  |  |  |  |  |  |  |  |  |  |  |
| **Set** | **Genotype or condition** | **Mean survival ± SEM (days)** | **Median survival (days)** | **75th percentile (days)** | **N dead  / initial N** | **Group** | **% Mean survival change  vs.  group a** | ***P* value  (log-rank) vs.  group a** | ***P* value (log-rank) vs.  group b** | ***P* value (log-rank) vs.  group c** | **Figure** |
| Pathway analysis | | | | | | | | | | | |
|  | wild type | 0.89 ± 0.02 | 0.87 | 1.00 | 139 / 139 | a |  |  |  |  | 7A |
|  | *daf-1(m40) IV* | 2.26 ± 0.05 | 2.28 | 2.67 | 125 / 125 | b | 153% | < 0.0001 |  |  |  |
|  | *tbh-1(ok1196) X* | 0.91 ± 0.01 | 0.87 | 1.02 | 139 / 139 | c | 2% | > 0.05 | < 0.0001 |  |  |
|  | *daf-1(m40) IV; tbh-1(ok1196) X* | 2.83 ± 0.04 | 2.83 | 3.10 | 142 / 142 | d | 217% | < 0.0001 | < 0.0001 | < 0.0001 |  |
|  | *daf-1(m40) IV* | 1.79 ± 0.05 | 1.73 | 2.11 | 94 / 94 | a |  |  |  |  | S7A |
|  | *tdc-1(ok914) II; daf-1(m40) IV* | 2.72 ± 0.05 | 2.62 | 3.05 | 128 / 128 | b | 52% | < 0.0001 |  |  |  |
| 1-day food-level conditioning | | | | | | | | | | | |
|  | 250 x 10^6^ *E. coli* OP50 lawn | 1.08 ± 0.02 | 1.09 | 1.24 | 107 / 121 | a |  |  |  |  | 7B |
|  | 50 x 10^6^ *E. coli* OP50 lawn | 1.06 ± 0.02 | 1.09 | 1.18 | 113 / 115 | b | -2% | > 0.05 |  |  |  |
|  | 10 x 10^6^ *E. coli* OP50 lawn | 0.93 ± 0.03 | 0.96 | 1.09 | 71 / 77 | c | -14% | 0.009 | 0.0009 |  |  |
|  | 0 *E. coli* OP50 lawn | 0.29 ± 0.02 | 0.30 | 0.37 | 56 / 56 | d | -73% | < 0.0001 | < 0.0001 | < 0.0001 |  |
| 2-day food-level conditioning | | | | | | | | | | | |
|  | 200 x 10^6^ *E. coli* OP50 lawn | 0.54 ± 0.01 | 0.52 | 0.61 | 141 / 148 | a |  |  |  |  | S7B |
|  | 100 x 10^6^ *E. coli* OP50 lawn | 0.56 ± 0.02 | 0.51 | 0.66 | 139 / 148 | b | 3% | > 0.05 |  |  |  |
|  | 50 x 10^6^ *E. coli* OP50 lawn | 0.48 ± 0.02 | 0.43 | 0.57 | 144 / 149 | c | -12% | 0.03 | 0.0009 |  |  |
|  | 25 x 10^6^ *E. coli* OP50 lawn | 0.21 ± 0.01 | 0.19 | 0.24 | 159 / 162 | d | -62% | < 0.0001 | < 0.0001 | < 0.0001 |  |
|  | with food | 0.73 ± 0.01 | 0.71 | 0.81 | 123 / 141 | a |  |  |  |  | 7C |
|  | no food | 0.12 ± 0.00 | 0.12 | 0.13 | 97 / 97 | b | -84% | < 0.0001 |  |  |  |
| Food ingestion | | | | | | | | | | | |
|  | wild type | 0.81 ± 0.01 | 0.79 | 0.93 | 152 / 152 | a |  |  |  |  | 7D |
|  | *eat-2(ad1116) II* | 0.62 ± 0.01 | 0.61 | 0.68 | 152 / 153 | b | -23% | < 0.0001 |  |  |  |
|  | *daf-3(mgDf90) X* | 0.84 ± 0.01 | 0.83 | 0.93 | 118 / 118 | c | 4% | > 0.05 | < 0.0001 |  |  |
|  | *eat-2(ad1116) II; daf-3(mgDf90) X* | 0.45 ± 0.01 | 0.45 | 0.49 | 128 / 128 | d | -44% | < 0.0001 | < 0.0001 | < 0.0001 |  |
