# Supplementary material for: Caenorhabditis elegans processes sensory information to choose between freeloading and self-defense strategies
Source: eLife. 2020 May 5;9:e56186. doi: 10.7554/eLife.56186 (PMC7213980; doi:10.7554/eLife.56186)
Supplement: Supplementary file 8. [file elife-56186-supp8.docx]

| **Supplementary file 8. Statistical analysis for Figure 8 and Figure 8—figure supplement 1.** | | | | | | | |  |  |  |  |  |
| --- | --- | --- | --- | --- | --- | --- | --- | --- | --- | --- | --- | --- |
|  |  |  |  |  |  |  |  |  |  |  |  |  |
| **Set** | **Worm genotype** | ***E. coli* genotype** | **Mean survival ± SEM (days)** | **Median survival (days)** | **75th percentile (days)** | **N dead  / initial N** | **Group** | **% Mean survival change  vs.  group a** | ***P* value  (log-rank) vs.  group a** | ***P* value (log-rank) vs.  group b** | ***P* value (log-rank) vs.  group c** | **Figure** |
| Bacterial genotype, 1 mM H_2_O_2_ | | | | | | | | | | | | |
|  | wild type | *katG katE ahpCF* | 1.42 ± 0.06 | 1.26 | 1.49 | 53 / 53 | a |  |  |  |  | 8B |
|  | *daf-7(ok3125) III* | *katG katE ahpCF* | 3.30 ± 0.21 | 3.25 | 3.46 | 25 / 25 | b | 132% | < 0.0001 |  |  |  |
|  | wild type | wild type | > 6.36 | . | . | 2 / 78 | c | > 347% | < 0.0001 | < 0.0001 |  |  |
|  | *daf-7(ok3125) III* | wild type | > 5.74 | . | . | 3 / 80 | d | > 304% | < 0.0001 | < 0.0001 | > 0.05 |  |
| Pathway analysis | | | | | | | | | | | | |
|  | wild type | *katG katE ahpCF* | 1.97 ± 0.04 | 1.96 | 2.31 | 137 / 149 | a |  |  |  |  | S8A |
|  | ASI: ablation | *katG katE ahpCF* | 2.57 ± 0.05 | 2.55 | 2.98 | 122 / 138 | b | 31% | < 0.0001 |  |  |  |
|  | wild type | *katG katE ahpCF* | 1.91 ± 0.06 | 1.74 | 2.43 | 126 / 126 | a |  |  |  |  | 8C |
|  | *wuIs151[ctl-1(+) + ctl-2(+) + ctl-3(+) + Pmro-3::GFP]* | *katG katE ahpCF* | 5.12 ± 0.19 | 5.18 | 6.45 | 96 / 96 | b | 168% | < 0.0001 |  |  |  |
|  | wild type | *katG katE ahpCF* | 1.14 ± 0.03 | 1.06 | 1.37 | 121 / 121 | a |  |  |  |  | 8D |
|  | *daf-1(m40) IV* | *katG katE ahpCF* | 2.70 ± 0.10 | 2.64 | 3.35 | 85 / 85 | b | 138% | < 0.0001 |  |  |  |
|  | *ctl-1(ok1242) II* | *katG katE ahpCF* | 0.91 ± 0.04 | 0.87 | 1.11 | 93 / 93 | c | -20% | 0.0002 | < 0.0001 |  |  |
|  | *ctl-1(ok1242) II; daf-1(m40) IV* | *katG katE ahpCF* | 1.51 ± 0.08 | 1.44 | 1.94 | 97 / 97 | d | 33% | < 0.0001 | < 0.0001 | < 0.0001 |  |
|  | wild type | *katG katE ahpCF* | 0.84 ± 0.03 | 0.81 | 1.01 | 110 / 110 | a |  |  |  |  | S8C |
|  | *daf-1(m40) IV* | *katG katE ahpCF* | 4.26 ± 0.19 | 3.78 | 5.19 | 109 / 109 | b | 408% | < 0.0001 |  |  |  |
|  | *ctl-2(ok1137) II* | *katG katE ahpCF* | 1.24 ± 0.04 | 1.16 | 1.43 | 112 / 112 | c | 48% | < 0.0001 | < 0.0001 |  |  |
|  | *ctl-2(ok1137) II; daf-1(m40) IV* | *katG katE ahpCF* | 4.24 ± 0.19 | 3.78 | 5.26 | 115 / 115 | d | 405% | < 0.0001 | > 0.05 | < 0.0001 |  |
