# Supplementary material for: Caenorhabditis elegans processes sensory information to choose between freeloading and self-defense strategies
Source: eLife. 2020 May 5;9:e56186. doi: 10.7554/eLife.56186 (PMC7213980; doi:10.7554/eLife.56186)
Supplement: Supplementary file 9. [file elife-56186-supp9.docx]

| **Supplementary file 9. Bacterial strains.** | |  |  |
| --- | --- | --- | --- |
|  |  |  |  |
| **Strain** | **Genotype** | **Source** | **Reference** |
| OP50 | *E. coli* B, uracil auxotroph | CGC | Brenner, 1974 |
| HT115 | *E. coli* F^-^ *mcrA, mcrB, IN(rrnD-rrnE)1, rnc14::Tn10(DE3 lysogen: lavUV5 promoter -T7 polymerase).* Tetracycline resistant | CGC | Kamath et al., 2001 |
| MG1655 | *E. coli* K12 F^-^ wild type | James Imlay | Seaver and Imlay, 2001 |
| JI377 | *E. coli* MG1655 *ahpCF katG katE* | James Imlay | Seaver and Imlay, 2001 |
