# Supplementary material for: Caenorhabditis elegans processes sensory information to choose between freeloading and self-defense strategies
Source: eLife. 2020 May 5;9:e56186. doi: 10.7554/eLife.56186 (PMC7213980; doi:10.7554/eLife.56186)
Supplement: Supplementary file 10. [file elife-56186-supp10.docx]

| **Supplementary file 10. *C. elegans* strains.** | |  |  |  |  |
| --- | --- | --- | --- | --- | --- |
|  |  |  |  |  |  |
| **Strain** | **Genotype** | **Comment** | **Figure** | **Source** | **Reference** |
| PR813 | *osm-5(p813) X* |  | 1 | CGC |  |
| PR671 | *tax-2(p671) I* |  | S1 | CGC |  |
| PY7512 | *tax-4(p678) III; Ex[Pttx-1::tax-4 + Punc-122::dsred]* |  | 1 | Piali Sengupta |  |
| FG540 | *udEx428[Psrh-124::ced- 3(p15), Psrh-142::ced- 3(p17), Psrh-142::gfp, Pelt-2::gfp]* | ADF ablation | 1 | Denise Ferkey | Krzyzanowski et al., 2016 |
| HCX179 | *bosEx179[Psre-1::csp-1b + Punc-122::GFP]* | ADL ablation | S1 | Howard Chang | Horspool and Chang, 2017 |
| SAY141 | *pgIs2[Pgcy-8::TU#813 + Pgcy-8::TU#814 + Punc-122::GFP + Pgcy-8::mCherry + Pgcy-8::GFP + Pttx-3::GFP]* | AFD ablation | 1 | CGC, This work | Glauser et al., 2011 |
| JPS271 | *vxEx265 [gcy-8p::ICE + myo-2p:mCherry].* | AFD ablation | S1 | CGC | Vidal-Gadea et al., 2015 |
| OH13098 | *che-1(ot75) I* | ASE fate determinant | 1 | CGC | Chang et al., 2003 |
| RJP3130 | *otEx3130Pops-1::p12 caspase + Pgcy-21 p17 caspase + Pmyo-3::RFP]; Is[Pets-5::mCherry + Pelt-2::GFP]* | ASG ablation | 1 | Roger Pocock | Juozaityte et al., 2017 |
| SAY113 | *Is[Psra-6::mCasp1 + Pmyo-3::RFP]* | ASH ablation | 1 | Takaaki Hirotsu | Yoshida et al., 2012 |
| PY7505 | *oyIs84 [Pgpa-4::TU#813 + Pgcy-27::TU#814 + Pgcy-27::GFP + Punc-122::DsRed]* | ASI ablation | 1, S8 | CGC | Beverly et al., 2011 |
| ZD763 | *mgIs40[Pdaf-28::nls-GFP]; jxEx102[Ptrx-1::ICE + Pofm-1::gfp]* | ASJ ablation | 1 | Dennis Kim | Cornils et al., 2011 |
| SAY119 | *qrIs2[Psra-9::mCasp1] 2x outcrossed* | ASK ablation | 1 | CGC, This work | Srinivasan et al., 2012 |
| SAY114 | *Ex[odr-10::mCasp1 + myo-3::GFP]* | AWA ablation | 1 | Takaaki Hirotsu | Yoshida et al., 2012 |
| SAY115 | *Is[str-1::mCasp1 + myo-3::GFP ]* | AWB ablation | 1 | Takaaki Hirotsu | Yoshida et al., 2012 |
| PY7502 | oyIs85[Pceh-36::TU#813 + Pceh-36::TU#814 + Psrtx-1::GFP + Punc-122::dsRed] | AWC ablation | 1 | CGC | Beverly et al., 2011 |
| LJ200 | *unc-119(ed3); ysIs1[punc-119cR + Pklp-6::ced-3 + Pklp-6:egl-1]* | IL2 ablation | 1 | Junho Lee | Lee et al., 2011 |
| ZD653 | *qdEx22[Pser-2::csp-1b; Pmyo-2::rfp]* | OLL ablation | 1 | Dennis Kim | Chang et al., 2011 |
| VM6365 | *lin-15(n765ts) X; akEx387[lin-15(+) + Pdat-1::gfp + Pdat-1::ICE]* | ADE/PDE/CEP ablation | 1 | Andres Maricq | Wragg et al., 2007 |
| JPS278 | *vxEx277[Pmec-3::ICE + Pmyo-2::mCherry]* | ALM/PLM/AVM/PVM/FLP/PVD ablation | 1 | CGC | Russell et al., 2014 |
| CX7102 | *lin-15B&lin-15A(n765) qaIs2241[Pgcy-36::egl-1 + Pgcy-35::GFP + lin-15(+)] X* | URX/AQR/PQR ablation | 1 | CGC | Chang et al., 2006 |
| RB2302 | *daf-7(ok3125) III* |  | 2,7, S2-S3 | CGC |  |
| ZD729 | *daf-7(ok3125) III; qdEx37[Pdaf-7::daf-7 + Pges-1::GFP]* |  | 2 | Dennis Kim | Fletcher and Kim, 2017 |
| ZD732 | *daf-7(ok3125) III; qdEx40[Ptrx-1::daf-7 + Pges-1::GFP]* |  | 2 | Dennis Kim | Fletcher and Kim, 2017 |
| ZD736 | *daf-7(ok3125) III; qdEx44[Pstr-3::daf-7 + Pges-1::GFP]* |  | 2 | Dennis Kim | Fletcher and Kim, 2017 |
| GR1311 | *daf-3(mgDf90) X* |  | 3 | CGC |  |
| SAY84 | *daf-3(mgDf90) X; oyIs84[Pgpa-4::TU#813 + Pgcy-27::TU#814 + Pgcy-27::GFP + Punc-122::DsRed]* |  | 3 | This work |  |
| SAY85 | *daf-1(m40) IV* 6x outcrossed |  | 2-7, S2-S3, S8 | This work |  |
| SAY72 | *daf-1(m40) IV; daf-3(mgDf90) X* |  | 3, S3 | This work |  |
| CB1372 | *daf-7(e1372) III* |  | 5, S2 | CGC |  |
| CB1376 | *daf-3(e1376) X* |  | S3 | CGC |  |
| ZD907 | *daf-7(ok3125) III; daf-3(e1376) X* |  | S3 | Dennis Kim | Fletcher and Kim, 2017 |
| CB1386 | *daf-5(e1386) II* |  | S3 | CGC |  |
| GR1278 | *daf-5(e1386) II; daf-7(e1372) III* |  | S3 | Jane Hubbard | Dalfo et al., 2012 |
| GC1149 | *daf-5(e1386) II; daf-1(m40) IV* |  | S3 | Jane Hubbard | Dalfo et al., 2012 |
| KQ275 | *daf-1(m40) IV; ftEx93[pglr-1::daf-1-gfp odr-1::dsRED]* |  | 3 | Young-Jai You | Greer et al., 2008 |
| KQ315 | *daf-1(m40) IV; ftEx166[pflp-1::daf-1::gfp + podr-1::dsRED]* |  | 3 | Young-Jai You | Greer et al., 2008 |
| KQ280 | *daf-1(m40) IV; ftEx98[Pdaf-1::daf-1-gfp + Podr-1::dsRED]* |  | 3 | Dennis Kim | Greer et al., 2008 |
| KQ251 | *daf-1(m40) IV; ftEx69[Pegl-3:: daf-1-gfp + Podr-1::dsRED]* |  | 3 | Dennis Kim | Greer et al., 2008 |
| KQ380 | *daf-1(m40) IV; ftEx205[Ptdc-1::daf-1-gfp + Podr-1::dsRED]* |  | 3 | Dennis Kim | Greer et al., 2008 |
| KQ265 | *daf-1(m40) IV; ftEx83[Posm-6::daf-1::GFP + Podr-1::dsRED]* |  | 3 | Dennis Kim | Greer et al., 2008 |
| KQ332 | *daf-1(m40) IV; ftEx183[pglr-7::daf-1-gfp odr-1::dsRED]* |  | S3 | Young-Jai You | Greer et al., 2008 |
| KQ256 | *daf-1(m40) IV; ftEx83[Pglr-8::daf-1::GFP + Podr-1::dsRED]* |  | S3 | Dennis Kim | Greer et al., 2008 |
| SAY137 | *daf-1(m40) IV; daf-3(mgDf90) X; witEx4[Pdaf-1::daf-3(+)-GFP + Punc-122::dsRED]* |  | 3 | This work |  |
| SAY118 | *daf-1(m40) IV; daf-3(mgDf90) X; witEx1[Ptdc-1::daf-3(+)::GFP+ Pmyo-2::RFP]* |  | S3 | This work |  |
| SAY133 | *daf-1(m40) IV; daf-3(mgDf90) X; witEx2[Ptdc-1::daf-3(+)::GFP + Pmyo-2::RFP]* |  | 3 | This work |  |
| SAY134 | *daf-1(m40) IV; daf-3(mgDf90) X; witEx3[Ptdc-1::daf-3(+)::GFP + Pmyo-2::RFP]* |  | S3 | This work |  |
| QZ117 | *daf-12(rh61rh411) X* |  | 4 | Joy Alcedo |  |
| GC1239 | *daf-7(e1372) III; daf-12(rh61rh411) X* |  | 4 | Jane Hubbard | Dalfo et al., 2012 |
| ZD1424 | *daf-1(m40) mgl-3(tm1766) IV; mgl-1(tm1811) X* |  | 4 | Dennis Kim | Fletcher and Kim, 2017 |
| SAY92 | *mgl-3(tm1766) IV; mgl-1(tm1811) X* |  | 4 | This work |  |
| SAY124 | *mgl-1(tm1811)X; daf-1(m40)IV* |  | S4 | This work |  |
| SAY130 | *mgl-1(tm1811) X* |  | S4 | This work |  |
| SAY128 | *mgl-3(tm1766) daf-1(m40) IV* |  | S4 | This work |  |
| SAY129 | *mgl-3(tm1766) IV* |  | S4 | This work |  |
| SAY80 | *daf-1(m40) IV; mes-1(ok2467) X* |  | 4 | This work |  |
| SAY20 | *mes-1(ok2467) X* 6x outcrossed |  | 4 | This work |  |
| SAY110 | *daf-3(mgDf90) mes-1(ok2467) X* |  | 4 | This work |  |
| QZ91 | *daf-2(e1370) III* 6x outcrossed |  | 5 | Joy Alcedo | Fernandes de Abreu et al., 2014 |
| SAY77 | *daf-2(e1370) III; daf-1(m40) IV* |  | 5 | This work |  |
| QZ80 | *ins-1(nr2091) IV* |  | S5 | Yun Zhang | Fernandes de Abreu et al., 2014 |
| QL28 | *ins-3(tm3608) II* |  | S5 | Yun Zhang | Fernandes de Abreu et al., 2014 |
| QL27 | *ins-4(tm3620) II* |  | 5 | Yun Zhang | Fernandes de Abreu et al., 2014 |
| QL24 | *ins-5(tm2560) II* |  | 5 | Yun Zhang | Fernandes de Abreu et al., 2014 |
| QZ81 | *ins-6(tm2416) II* |  | 5 | Yun Zhang | Fernandes de Abreu et al., 2014 |
| QZ83 | *daf-28(tm2308) V* |  | 5 | Yun Zhang | Fernandes de Abreu et al., 2014 |
| QZ60 | *daf-16(mu86) I* |  | 5 | Joy Alcedo | Fernandes de Abreu et al., 2014 |
| QZ218 | *daf-16(mu86) I; daf-2(e1370) III* |  | 5 | Joy Alcedo | Fernandes de Abreu et al., 2014 |
| GR1307 | *daf-16(mgDf50) I* |  | 5 | CGC |  |
| LRB209 | *ins-4 ins-5 ins-6(hpDf761) II; ayIs7 IV; daf-28(tm2308) V* |  | 5 | Ryan Baugh | Kaplan et al., 2015 |
| LRB228 | *daf-16(mgDf50) I; ins-4 ins-5 ins-6(hpDf761) II; ayIs7 IV; daf-28(tm2308) V* |  | 5 | Ryan Baugh | Kaplan et al., 2015 |
| GC1248 | *daf-16(mu86) I; daf-7(e1372) III* |  | 5 | Jane Hubbard | Dalfo et al., 2012 |
| SAY74 | *daf-16(mu86) I; daf-1(m40) IV* |  | 5 | This work |  |
| SAY186 | *daf-1(m40) hlh-30(tm1978) IV* |  | S5 | This work |  |
| SAY102 | *daf-16(mu86) I; daf-1(m40) IV; qyIs288[Pdaf-16::GFP::daf-16 + Punc-119(+)]* |  | 5 | This work |  |
| SAY103 | *daf-16(mu86) I; daf-1(m40) IV; unc-119(ed4) III; qyEx264[Pmyo-3::GFP::daf-16a + unc-119(+)]* |  | 5 | This work |  |
| SAY104 | *daf-16(mu86) I; daf-1(m40) IV; qyIs290[Pcol-12::GFP::daf-16 + unc-119(+)]* |  | 5 | This work |  |
| SAY105 | *daf-16(mu86) I; daf-1(m40) IV; qyIs292[Pges-1::GFP::daf-16]* |  | 5 | This work |  |
| SAY100 | *daf-16(mu86) I; daf-1(m40) IV; qyIs294[Punc-119::GFP::daf-16]* |  | 5 | This work |  |
| SAY65 | *tbh-1(ok1196) X* |  | 7 | This work |  |
| KQ364 | *daf-1(m40) IV; tbh-1(ok1196) X* |  | 7 | Dennis Kim | Fletcher and Kim, 2017 |
| SAY64 | *daf-1(m40) IV; tdc-1(ok914) II* |  | S7 | Dennis Kim | Fletcher and Kim, 2017 |
| QZ414 | *eat-2(ad1116) II* |  | 7 | Joy Alcedo |  |
| SAY89 | *eat-2(ad1116) II; daf-3(mgDf90) X* |  | 7 | This work |  |
| GA800 | *wuIs151[ctl-1(+) + ctl-2(+) + ctl-3(+) + Pmro-3::GFP]* |  | 8 | CGC |  |
| UN1781 | *ctl-1(ok1242) II* |  | 8 | Erin Cram |  |
| SAY200 | *ctl-1(ok1242) II; daf-1(m40) IV* |  | 8 | This work |  |
| UN18129 | *ctl-2(ok1137) II* |  | S8 | Erin Cram |  |
| SAY191 | *ctl-2(ok1137) II; daf-1(m40) IV* |  | S8 | This work |  |
| KHA166 | *chuIs166[unc-119(+), Pctl-1::Bxy-ctl-1::gfp]* |  | 8 | Koichi Hasegawa | Hamaguchi et al., 2019 |
| SAY231 | *daf-1(m40) IV; chuIs166[unc-119(+), Pctl-1::Bxy-ctl-1::gfp]* |  | 8 | This work |  |
