# Supplementary material for: Caenorhabditis elegans processes sensory information to choose between freeloading and self-defense strategies
Source: eLife. 2020 May 5;9:e56186. doi: 10.7554/eLife.56186 (PMC7213980; doi:10.7554/eLife.56186)
Supplement: Supplementary file 11. [file elife-56186-supp11.docx]

| **Supplementary file 11. PCR genotyping primers and enzymes, and phenotypes used for strain construction.** | | | |
| --- | --- | --- | --- |
|  |  |  |  |
| **Allele** | **phenotype** | **Primers** | **Restriction Enzyme** |
| *daf-3(mgDf90)* | - | AAACGCGTCATGTGGACCAC | - |
|  |  | ACCCTCATGCCTACTGTCAG |  |
| *oyIs84* | fluorescent marker | - | - |
| *daf-1(m40)* | dauer constitutive | GAGACGATCATCCACTTGGTAAG | Tsp45I |
|  |  | AAATCTTCCGGATCCAACTCTAC |  |
| *daf-7(ok3125)* | dauer constitutive | ATCTTCACTCCCGGTGTTTATCT | *-* |
|  |  | CTCAGGATTGGAGACTTTGTGAG |  |
| *mes-1(ok2467)* | sterile | - | *-* |
| *tdc-1(ok914)* | - | AACGGTGCATTTTTCAGGAC | *-* |
|  |  | GGACGTTGAGAATGCGAAAT |  |
|  |  | AAATGGTTTACGGGCTTGG |  |
|  |  | ATGGTTGGCCATGTTGAGAT |  |
| *eat-2(ad1116)* | decreased pumping | GGAGCCACTTAGGACACC | Hpy188I |
|  |  | CCACACTATCTTTCTACCAC |  |
| *tbh-1(ok1196)* | - | AAGCAGGATCAGGAGCACAT | *-* |
|  |  | ATGAGAAGTGCCGTTGCTCT |  |
|  |  | CATGTCATTGATGGCTGGAC |  |
|  |  | GAACGCCAGTTGGTTGATTT |  |
| *daf-2(e1370)* | dauer constitutive | GACGATCCCGAGGTGAGTAT | *-* |
|  |  | CAGCGATGGTTGTGATGGAA |  |
| *daf-16(mu86)* | - | AGAACACCATGGGGGCACTGGAT | *-* |
|  |  | GGCGGGAATGAAGCAAGAGCCAA |  |
|  |  | TGACGCTCACCTTGAAAAGGTCAAT |  |
|  |  | GGAACCGATTCGCCAACCCATGA |  |
| *ins-3(tm3608)* | - | GATCAGCATTGTCACCTGAC | *-* |
|  |  | TGGCAACTGATGTCCGGTAT |  |
| *ins-4(tm3620)* | - | CCGCCCAATCCCTTTAACGT | *-* |
|  |  | GATGGCTTGTTGGACGACTG |  |
|  |  | TGGCGCTTGACGCATCAGTC |  |
|  |  | GAGTGCACTGTGTTGTGCAG |  |
| *ins-5(tm2560)* | - | GGCTCCTTGCGCCATGATGT | *-* |
|  |  | ATCCTTGAATGCCCGTGAGT |  |
|  |  | GCTCCTCCCATGTTGGATTG |  |
|  |  | GGTTTCAGGAGGGTGACGGT |  |
| *ins-6(tm2416)* | - | TTTACCCACCCCTTCGTGAT | *-* |
|  |  | TTGTACAAGCCACTGGGATG |  |
|  |  | TCGTGATGCTCCGCCTATTG |  |
|  |  | ACAGAGACTGATATCGGAGT |  |
| *daf-28(tm2308)* | - | TCCGCCCACTTTGAGCTATA | *-* |
|  |  | GCACCCGATCTGACGACACT |  |
|  |  | GGGTTATCACTAGGAAGTTG |  |
|  |  | ACCGAGAGGTAGGGGTAATT |  |
| *hlh-30(tm1978)* | - | TGTCACCTCGGATAGGAAGCCGG | - |
|  |  | TGGCGGGAAGTTCGAAAATTGTTGA |  |
|  |  | GCTGAAATGTTTGCTCAAAAGCGCC |  |
| *ctl-2(ok1137)* | - | TACCCAGAAGCGTAATCCACAG | *-* |
|  |  | CATCTTGTCTGGCGAGAACTCG |  |
