# Supplementary material for: Caenorhabditis elegans processes sensory information to choose between freeloading and self-defense strategies
Source: eLife. 2020 May 5;9:e56186. doi: 10.7554/eLife.56186 (PMC7213980; doi:10.7554/eLife.56186)
Supplement: Supplementary file 12. [file elife-56186-supp12.docx]

| **Key Resources Table** | | | | |
| --- | --- | --- | --- | --- |
| **Reagent type (species) or resource** | **Designation** | **Source or reference** | **Identifiers** | **Additional information** |
| Strain, strain background(*C. elegans*) | PR813 | CGC | WormBase ID: WBStrain00030797 | Genotype: *osm-5(p813) X* |
| Strain, strain background(*C. elegans*) | PR671 | CGC | WormBase ID: WBStrain00030780 | Genotype: *tax-2(p671) I* |
| Strain, strain background(*C. elegans*) | PY7512 | Piali Sengupta (Brandeis University)  ([Beverly et al., 2011](#_ENREF_13)) |  | Genotype: *tax-4(p678) III; Ex[Pttx-1::tax-4 + Punc-122::dsred]* |
| Strain, strain background(*C. elegans*) | FG540 | Denise Ferkey (University at Buffalo)  ([Krzyzanowski et al., 2016](#_ENREF_62)) |  | ADF ablation. Genotype: *udEx428[Psrh-124::ced- 3(p15), Psrh-142::ced- 3(p17), Psrh-142::gfp, Pelt-2::gfp]* |
| Strain, strain background(*C. elegans*) | HCX179 | Howard Chang (Binghamton University)  ([Horspool and Chang, 2017](#_ENREF_48)) |  | ADL ablation. Genotype: *bosEx179[Psre-1::csp-1b + Punc-122::GFP]* |
| Strain, strain background(*C. elegans*) | SAY141 | CGC.  ([Glauser et al., 2011](#_ENREF_42)) |  | AFD ablation. Genotype: *pgIs2[Pgcy-8::TU#813 + Pgcy-8::TU#814 + Punc-122::GFP + Pgcy-8::mCherry + Pgcy-8::GFP + Pttx-3::GFP]* |
| Strain, strain background(*C. elegans*) | JPS271 | CGC.  ([Vidal-Gadea et al., 2015](#_ENREF_121)) | WormBase ID: WBStrain00022708 | AFD ablation. Genotype: *vxEx265[gcy-8p::ICE + myo-2p:mCherry].* |
| Strain, strain background(*C. elegans*) | OH13098 | CGC.  ([Chang et al., 2003](#_ENREF_22)) | WormBase ID: WBStrain00029712 | ASE fate determinant. Genotype: *che-1(ot75) I* |
| Strain, strain background(*C. elegans*) | RJP3130 | Roger Pocock (Monash University)  ([Juozaityte et al., 2017](#_ENREF_54)) |  | ASG ablation. Genotype: *otEx3130[Pops-1::p12 caspase + Pgcy-21 p17 caspase + Pmyo-3::RFP]; Is[Pets-5::mCherry + Pelt-2::GFP]* |
| Strain, strain background(*C. elegans*) | SAY113 | Takaaki Hirotsu (University of Tokyo)  ([Yoshida et al., 2012](#_ENREF_128)) |  | ASH ablation. Genotype: *Is[Psra-6::mCasp1 + Pmyo-3::RFP]* |
| Strain, strain background(*C. elegans*) | PY7505 | CGC.  ([Beverly et al., 2011](#_ENREF_13)) | WormBase ID: WBStrain00031144 | ASI ablation. Genotype: *oyIs84 [Pgpa-4::TU#813 + Pgcy-27::TU#814 + Pgcy-27::GFP + Punc-122::DsRed]* |
| Strain, strain background(*C. elegans*) | ZD763 | Dennis Kim (Boston Children’s Hospital)  ([Cornils et al., 2011](#_ENREF_27)) |  | ASJ ablation. Genotype: *mgIs40[Pdaf-28::nls-GFP]; jxEx102[Ptrx-1::ICE + Pofm-1::gfp]* |
| Strain, strain background(*C. elegans*) | SAY119 | CGC.  ([Srinivasan et al., 2012](#_ENREF_108)) | WormBase ID: WBStrain00030970 | ASK ablation. Genotype: *qrIs2[Psra-9::mCasp1]* 2x outcrossed |
| Strain, strain background(*C. elegans*) | SAY114 | Takaaki Hirotsu (University of Tokyo)  ([Yoshida et al., 2012](#_ENREF_128)) |  | AWA ablation. Genotype: *Ex[odr-10::mCasp1 + myo-3::GFP]* |
| Strain, strain background(*C. elegans*) | SAY115 | Takaaki Hirotsu (University of Tokyo)  ([Yoshida et al., 2012](#_ENREF_128)) |  | AWB ablation. Genotype: *Is[str-1::mCasp1 + myo-3::GFP ]* |
| Strain, strain background(*C. elegans*) | PY7502 | CGC.  ([Beverly et al., 2011](#_ENREF_13)) | WormBase ID: WBStrain00031143 | AWC ablation. Genotype: *oyIs85[Pceh-36::TU#813 + Pceh-36::TU#814 + Psrtx-1::GFP + Punc-122::dsRed]* |
| Strain, strain background(*C. elegans*) | LJ200 | Junho Lee (Seoul National University)  ([Lee et al., 2011](#_ENREF_64)) |  | IL2 ablation. Genotype: *unc-119(ed3); ysIs1[punc-119cR + Pklp-6::ced-3 + Pklp-6:egl-1]* |
| Strain, strain background(*C. elegans*) | ZD653 | Dennis Kim (Boston Children’s Hospital)  ([Chang et al., 2011](#_ENREF_21)) |  | OLL ablation. Genotype: *qdEx22[Pser-2::csp-1b; Pmyo-2::rfp]* |
| Strain, strain background(*C. elegans*) | VM6365 | Andres Maricq (University of Utah)  ([Wragg et al., 2007](#_ENREF_125)) |  | ADE/PDE/CEP ablation. Genotype: *lin-15(n765ts) X; akEx387[lin-15(+) + Pdat-1::gfp + Pdat-1::ICE]* |
| Strain, strain background(*C. elegans*) | JPS278 | CGC.  ([Russell et al., 2014](#_ENREF_104)) | WormBase ID: WBStrain00022709 | ALM/PLM/AVM/PVM/FLP/PVD ablation. Genotype:*vxEx277[Pmec-3::ICE + Pmyo-2::mCherry]* |
| Strain, strain background(*C. elegans*) | CX7102 | CGC.  ([Chang et al., 2006](#_ENREF_20)) | WormBase ID: WBStrain00005284 | URX/AQR/PQR ablation. Genotype: *lin-15B&lin-15A(n765) qaIs2241[Pgcy-36::egl-1 + Pgcy-35::GFP + lin-15(+)] X* |
| Strain, strain background(*C. elegans*) | RB2302 | CGC | WormBase ID: WBStrain00032980 | Genotype: *daf-7(ok3125) III* |
| Strain, strain background(*C. elegans*) | ZD729 | Dennis Kim (Boston Children’s Hospital)  ([Fletcher and Kim, 2017](#_ENREF_38)) |  | Genotype: *daf-7(ok3125) III; qdEx37[Pdaf-7::daf-7 + Pges-1::GFP]* |
| Strain, strain background(*C. elegans*) | ZD732 | Dennis Kim (Boston Children’s Hospital)  ([Fletcher and Kim, 2017](#_ENREF_38)) |  | Genotype: *daf-7(ok3125) III; qdEx40[Ptrx-1::daf-7 + Pges-1::GFP]* |
| Strain, strain background(*C. elegans*) | ZD736 | Dennis Kim (Boston Children’s Hospital)  ([Fletcher and Kim, 2017](#_ENREF_38)) |  | Genotype: *daf-7(ok3125) III; qdEx44[Pstr-3::daf-7 + Pges-1::GFP]* |
| Strain, strain background(*C. elegans*) | GR1311 | CGC | WormBase ID: WBStrain00007899 | Genotype: *daf-3(mgDf90) X* |
| Strain, strain background(*C. elegans*) | SAY84 | This study |  | Genotype: *daf-3(mgDf90) X; oyIs84[Pgpa-4::TU#813 + Pgcy-27::TU#814 + Pgcy-27::GFP + Punc-122::DsRed].*  Reagent requests: see Materials and methods |
| Strain, strain background(*C. elegans*) | SAY85 | This study. |  | Genotype: *daf-1(m40) IV* 6x outcrossed. Reagent requests: see Materials and methods |
| Strain, strain background(*C. elegans*) | SAY72 | This study |  | Genotype: *daf-1(m40) IV; daf-3(mgDf90) X*  Reagent requests: see Materials and methods |
| Strain, strain background(*C. elegans*) | CB1372 | CGC | WormBase ID: WBStrain00004310 | Genotype: *daf-7(e1372) III* |
| Strain, strain background(*C. elegans*) | CB1376 | CGC | WormBase ID: WBStrain00004312 | Genotype: *daf-3(e1376) X* |
| Strain, strain background(*C. elegans*) | ZD907 | Dennis Kim (Boston Children’s Hospital)  ([Fletcher and Kim, 2017](#_ENREF_38)) |  | Genotype: *daf-7(ok3125) III; daf-3(e1376) X* |
| Strain, strain background(*C. elegans*) | CB1386 | CGC | WormBase ID: WBStrain00004317 | Genotype: *daf-5(e1386) II* |
| Strain, strain background(*C. elegans*) | GR1278 | Jane Hubbard (New York University)  ([Dalfo et al., 2012](#_ENREF_30)) |  | Genotype: *daf-5(e1386) II; daf-7(e1372) III* |
| Strain, strain background(*C. elegans*) | GC1149 | Jane Hubbard (New York University)  ([Dalfo et al., 2012](#_ENREF_30)) |  | Genotype: *daf-5(e1386) II; daf-1(m40) IV* |
| Strain, strain background(*C. elegans*) | KQ275 | Young-Jai You  (Nagoya University)  ([Greer et al., 2008](#_ENREF_44)) |  | Genotype: *daf-1(m40) IV; ftEx93[pglr-1::daf-1-gfp odr-1::dsRED]* |
| Strain, strain background(*C. elegans*) | KQ315 | Young-Jai You  (Nagoya University)  ([Greer et al., 2008](#_ENREF_44)) |  | Genotype: *daf-1(m40) IV; ftEx166[pflp-1::daf-1::gfp + podr-1::dsRED]* |
| Strain, strain background(*C. elegans*) | KQ280 | Dennis Kim (Boston Children’s Hospital)  ([Greer et al., 2008](#_ENREF_44)) |  | Genotype: *daf-1(m40) IV; ftEx98[Pdaf-1::daf-1-gfp + Podr-1::dsRED]* |
| Strain, strain background(*C. elegans*) | KQ251 | Dennis Kim (Boston Children’s Hospital)  ([Greer et al., 2008](#_ENREF_44)) |  | Genotype: *daf-1(m40) IV; ftEx69[Pegl-3:: daf-1-gfp + Podr-1::dsRED]* |
| Strain, strain background(*C. elegans*) | KQ380 | Dennis Kim (Boston Children’s Hospital)  ([Greer et al., 2008](#_ENREF_44)) |  | Genotype: *daf-1(m40) IV; ftEx205[Ptdc-1::daf-1-gfp + Podr-1::dsRED]* |
| Strain, strain background(*C. elegans*) | KQ265 | Dennis Kim (Boston Children’s Hospital)  ([Greer et al., 2008](#_ENREF_44)) |  | Genotype: *daf-1(m40) IV; ftEx83[Posm-6::daf-1::GFP + Podr-1::dsRED]* |
| Strain, strain background(*C. elegans*) | KQ332 | Young-Jai You  (Nagoya University)  ([Greer et al., 2008](#_ENREF_44)) |  | Genotype: *daf-1(m40) IV; ftEx183[pglr-7::daf-1-gfp odr-1::dsRED]* |
| Strain, strain background(*C. elegans*) | KQ256 | Dennis Kim (Boston Children’s Hospital)  ([Greer et al., 2008](#_ENREF_44)) |  | Genotype: *daf-1(m40) IV; ftEx83[Pglr-8::daf-1::GFP + Podr-1::dsRED]* |
| Strain, strain background(*C. elegans*) | SAY137 | This study |  | Genotype: *daf-1(m40) IV; daf-3(mgDf90) X; witEx4[Pdaf-1::daf-3(+)-GFP + Punc-122::dsRED]*  Reagent requests: see Materials and methods |
| Strain, strain background(*C. elegans*) | SAY118 | This study |  | Genotype: *daf-1(m40) IV; daf-3(mgDf90) X; witEx1[Ptdc-1::daf-3(+)::GFP+ Pmyo-2::RFP]*  Reagent requests: see Materials and methods |
| Strain, strain background(*C. elegans*) | SAY133 | This study |  | Genotype: *daf-1(m40) IV; daf-3(mgDf90) X; witEx2[Ptdc-1::daf-3(+)::GFP + Pmyo-2::RFP]*  Reagent requests: see Materials and methods |
| Strain, strain background(*C. elegans*) | SAY134 | This study |  | Genotype: *daf-1(m40) IV; daf-3(mgDf90) X; witEx3[Ptdc-1::daf-3(+)::GFP + Pmyo-2::RFP]*  Reagent requests: see Materials and methods |
| Strain, strain background(*C. elegans*) | QZ117 | Joy Alcedo (Wayne State University) |  | Genotype: *daf-12(rh61rh411) X* |
| Strain, strain background(*C. elegans*) | GC1239 | Jane Hubbard (New York University)  ([Dalfo et al., 2012](#_ENREF_30)) |  | Genotype: *daf-7(e1372) III; daf-12(rh61rh411) X* |
| Strain, strain background(*C. elegans*) | ZD1424 | Dennis Kim (Boston Children’s Hospital)  ([Fletcher and Kim, 2017](#_ENREF_38)) |  | Genotype: *daf-1(m40) mgl-3(tm1766) IV; mgl-1(tm1811) X* |
| Strain, strain background(*C. elegans*) | SAY92 | This study |  | Genotype: *mgl-3(tm1766) IV; mgl-1(tm1811) X*  Reagent requests: see Materials and methods |
| Strain, strain background(*C. elegans*) | SAY124 | This study |  | Genotype: *mgl-1(tm1811)X; daf-1(m40)IV*  Reagent requests: see Materials and methods |
| Strain, strain background(*C. elegans*) | SAY130 | This study |  | Genotype: *mgl-1(tm1811) X*  Reagent requests: see Materials and methods |
| Strain, strain background(*C. elegans*) | SAY128 | This study |  | Genotype: *mgl-3(tm1766) daf-1(m40) IV*  Reagent requests: see Materials and methods |
| Strain, strain background(*C. elegans*) | SAY129 | This study |  | Genotype: *mgl-3(tm1766) IV*  Reagent requests: see Materials and methods |
| Strain, strain background(*C. elegans*) | SAY80 | This study |  | Genotype: *daf-1(m40) IV; mes-1(ok2467) X*  Reagent requests: see Materials and methods |
| Strain, strain background(*C. elegans*) | SAY20 | This study |  | Genotype: *mes-1(ok2467) X* 6x outcrossed  Reagent requests: see Materials and methods |
| Strain, strain background(*C. elegans*) | SAY110 | This study |  | Genotype: *daf-3(mgDf90) mes-1(ok2467) X*  Reagent requests: see Materials and methods |
| Strain, strain background(*C. elegans*) | QZ91 | Joy Alcedo (Wayne State University)  ([Fernandes de Abreu et al., 2014](#_ENREF_37)) |  | Genotype: *daf-2(e1370) III* 6x outcrossed |
| Strain, strain background(*C. elegans*) | SAY77 | This study |  | Genotype: *daf-2(e1370) III; daf-1(m40) IV*  Reagent requests: see Materials and methods |
| Strain, strain background(*C. elegans*) | QZ80 | Yun Zhang (Harvard University)  ([Fernandes de Abreu et al., 2014](#_ENREF_37)) |  | Genotype: *ins-1(nr2091) IV* |
| Strain, strain background(*C. elegans*) | QL28 | Yun Zhang (Harvard University)  ([Fernandes de Abreu et al., 2014](#_ENREF_37)) |  | Genotype: *ins-3(tm3608) II* |
| Strain, strain background(*C. elegans*) | QL27 | Yun Zhang (Harvard University)  ([Fernandes de Abreu et al., 2014](#_ENREF_37)) |  | Genotype: *ins-4(tm3620) II* |
| Strain, strain background(*C. elegans*) | QL24 | Yun Zhang (Harvard University)  ([Fernandes de Abreu et al., 2014](#_ENREF_37)) |  | Genotype: *ins-5(tm2560) II* |
| Strain, strain background(*C. elegans*) | QZ81 | Yun Zhang (Harvard University)  ([Fernandes de Abreu et al., 2014](#_ENREF_37)) |  | Genotype: *ins-6(tm2416) II* |
| Strain, strain background(*C. elegans*) | QZ83 | Yun Zhang (Harvard University)  ([Fernandes de Abreu et al., 2014](#_ENREF_37)) |  | Genotype: *daf-28(tm2308) V* |
| Strain, strain background(*C. elegans*) | QZ60 | Joy Alcedo (Wayne State University)  ([Fernandes de Abreu et al., 2014](#_ENREF_37)) |  | Genotype: *daf-16(mu86) I* |
| Strain, strain background(*C. elegans*) | QZ218 | Joy Alcedo (Wayne State University)  ([Fernandes de Abreu et al., 2014](#_ENREF_37)) |  | Genotype: *daf-16(mu86) I; daf-2(e1370) III* |
| Strain, strain background(*C. elegans*) | GR1307 | CGC | WormBase ID: WBStrain00007895 | Genotype: *daf-16(mgDf50) I* |
| Strain, strain background(*C. elegans*) | LRB209 | Ryan Baugh (Duke University)  ([Kaplan et al., 2015](#_ENREF_57)) |  | Genotype: *ins-4 ins-5 ins-6(hpDf761) II; ayIs7 IV; daf-28(tm2308) V* |
| Strain, strain background(*C. elegans*) | LRB228 | Ryan Baugh (Duke University)  ([Kaplan et al., 2015](#_ENREF_57)) |  | Genotype: *daf-16(mgDf50) I; ins-4 ins-5 ins-6(hpDf761) II; ayIs7 IV; daf-28(tm2308) V* |
| Strain, strain background(*C. elegans*) | GC1248 | Jane Hubbard (New York University)  ([Kaplan et al., 2015](#_ENREF_57)) |  | Genotype: *daf-16(mu86) I; daf-7(e1372) III* |
| Strain, strain background(*C. elegans*) | SAY74 | This study |  | Genotype: *daf-16(mu86) I; daf-1(m40) IV*  Reagent requests: see Materials and methods |
| Strain, strain background(*C. elegans*) | SAY186 | This study |  | Genotype: *daf-1(m40) hlh-30(tm1978) IV*  Reagent requests: see Materials and methods |
| Strain, strain background(*C. elegans*) | SAY102 | This study |  | Genotype: *daf-16(mu86) I; daf-1(m40) IV; qyIs288[Pdaf-16::GFP::daf-16 + Punc-119(+)]*  Reagent requests: see Materials and methods |
| Strain, strain background(*C. elegans*) | SAY103 | This study |  | Genotype: *daf-16(mu86) I; daf-1(m40) IV; unc-119(ed4) III; qyEx264[Pmyo-3::GFP::daf-16a + unc-119(+)]*  Reagent requests: see Materials and methods |
| Strain, strain background(*C. elegans*) | SAY104 | This study |  | Genotype: *daf-16(mu86) I; daf-1(m40) IV; qyIs290[Pcol-12::GFP::daf-16 + unc-119(+)]*  Reagent requests: see Materials and methods |
| Strain, strain background(*C. elegans*) | SAY105 | This study |  | Genotype: *daf-16(mu86) I; daf-1(m40) IV; qyIs292[Pges-1::GFP::daf-16]*  Reagent requests: see Materials and methods |
| Strain, strain background(*C. elegans*) | SAY100 | This study |  | Genotype: *daf-16(mu86) I; daf-1(m40) IV; qyIs294[Punc-119::GFP::daf-16]*  Reagent requests: see Materials and methods |
| Strain, strain background(*C. elegans*) | SAY65 | This study |  | Genotype: *tbh-1(ok1196) X*  Reagent requests: see Materials and methods |
| Strain, strain background(*C. elegans*) | KQ364 | Dennis Kim (Boston Children’s Hospital)  ([Fletcher and Kim, 2017](#_ENREF_38)) |  | Genotype: *daf-1(m40) IV; tbh-1(ok1196) X* |
| Strain, strain background(*C. elegans*) | SAY64 | Dennis Kim (Boston Children’s Hospital)  ([Fletcher and Kim, 2017](#_ENREF_38)) |  | Genotype: *daf-1(m40) IV; tdc-1(ok914) II* |
| Strain, strain background(*C. elegans*) | QZ414 | Joy Alcedo (Wayne State University) |  | Genotype: *eat-2(ad1116) II* |
| Strain, strain background(*C. elegans*) | SAY89 | This study |  | Genotype: *eat-2(ad1116) II; daf-3(mgDf90) X*  Reagent requests: see Materials and methods |
| Strain, strain background(*C. elegans*) | GA800 | CGC | WormBase ID: WBStrain00007677 | Genotype: *wuIs151[ctl-1(+) + ctl-2(+) + ctl-3(+) + Pmro-3::GFP]* |
| Strain, strain background(*C. elegans*) | UN1781 | Erin Cram (Northeastern University) |  | Genotype: *ctl-1(ok1242) II* |
| Strain, strain background(*C. elegans*) | SAY200 | This study |  | Genotype: *ctl-1(ok1242) II; daf-1(m40) IV*  Reagent requests: see Materials and methods |
| Strain, strain background(*C. elegans*) | UN18129 | Erin Cram (Northeastern University) |  | Genotype: *ctl-2(ok1137) II* |
| Strain, strain background(*C. elegans*) | SAY191 | This study |  | Genotype: *ctl-2(ok1137) II; daf-1(m40) IV*  Reagent requests: see Materials and methods |
| Strain, strain background(*C. elegans*) | KHA166 | Koichi Hasegawa (Chubu University)  ([Hamaguchi et al., 2019](#_ENREF_46)) |  | Genotype: *chuIs166[unc-119(+), Pctl-1::Bxy-ctl-1::gfp]* |
| Strain, strain background(*C. elegans*) | SAY231 | This study |  | Genotype: *daf-1(m40) IV; chuIs166[unc-119(+), Pctl-1::Bxy-ctl-1::gfp]*  Reagent requests: see Materials and methods |
| Software | Lifespan Machine | ([Stroustrup et al., 2013](#_ENREF_111)) |  |  |
| Strain, strain background(*E. coli*) | OP50 | CGC.  ([Brenner, 1974](#_ENREF_15)) | WormBase ID: WBStrain00041969 | Genotype: *E. coli* B, uracil auxotroph |
| Strain, strain background(*E. coli*) | HT115 | CGC.  ([Kamath et al., 2001](#_ENREF_55)) | WormBase ID: WBStrain00041079 | Genotype: *E. coli* B, uracil auxotroph |
| Strain, strain background(*E. coli*) | MG1655 | James Imlay (University of Illinois, Urbana-Champaign)  ([Seaver and Imlay, 2001](#_ENREF_106)) | *E. coli* Genetic Stock Center #: 6300 | Genotype: *E. coli* K12 F^-^ wild type |
| Strain, strain background(*E. coli*) | JI377 | James Imlay (University of Illinois, Urbana-Champaign)  ([Seaver and Imlay, 2001](#_ENREF_106)) |  | Genotype: *E. coli* MG1655 *ahpCF katG katE* |
| Sequence-based reagent | *ctl-1* RT-PCR primers | This study. |  | TTCCATTTCAAGCCTGCTC |
|  |  |  |  | ATAGTCTGGATCCGAAGAGG |
| Sequence-based reagent | *rpl-32* RT-PCR primers | ([Amrit et al., 2019](#_ENREF_2)) |  | GGATTTGGACATGCTCCTC |
|  |  |  |  | GATTCCCTTGCGGCTCTT |
| Sequence-based reagent | *daf-3(mgDf90)* genotyping primers | Protocol from Kaveh Ashrafi (Universtiy of California, San Francisco) |  | AAACGCGTCATGTGGACCAC |
|  |  |  |  | ACCCTCATGCCTACTGTCAG |
| Sequence-based reagent | *daf-1(m40)* genotyping primers | This study.  Tsp45I from New England BioLabs | Tsp45I cat #: R0583S | GAGACGATCATCCACTTGGTAAG |
|  |  |  |  | AAATCTTCCGGACCAACTCTAC |
|  |  |  |  | Tsp45I digestion |
| Sequence-based reagent | *daf-7(ok3125)* genotyping primers | This study |  | ATCTTCACTCCCGGTGTTTATCT |
|  |  |  |  | CTCAGGATTGGAGACTTTGTGAG |
| Sequence-based reagent | *tdc-1(ok914)* genotyping primers | *C. elegans* Gene Knockout Consortium |  | AACGGTGCATTTTTCAGGAC |
|  |  |  |  | GGACGTTGAGAATGCGAAAT |
|  |  |  |  | AAATGGTTTACGGGCTTGG |
|  |  |  |  | ATGGTTGGCCATGTTGAGAT |
| Sequence-based reagent | *eat-2(ad1116)* genotyping primers | Protocol from Joy Alcedo (Wayne State University)  Hpy188I from New England BioLabs | Hpy188I cat #: R0617S | GGAGCCACTTAGGACACC |
|  |  |  |  | CCACACTATCTTTCTACCAC |
|  |  |  |  | Hpy188I digestion |
| Sequence-based reagent | *tbh-1(ok1196)* genotyping primers | *C. elegans* Gene Knockout Consortium |  | AAGCAGGATCAGGAGCACAT |
|  |  |  |  | ATGAGAAGTGCCGTTGCTCT |
|  |  |  |  | CATGTCATTGATGGCTGGAC |
|  |  |  |  | GAACGCCAGTTGGTTGATTT |
| Sequence-based reagent | *daf-2(e1370)* genotyping primers | This study |  | CACCTCATCATTACTCAAACCAATATAGGG |
|  |  |  |  | TATGAAATGGTTACACTCGGTGCTCCGT |
|  |  |  |  | GGTGAGTATCTCCAGCACATTTTCATCA |
|  |  |  |  | CGGAATGGCTCGTGATCTATTCTATCAT |
| Sequence-based reagent | *daf-16(mu86)* genotyping primers | This study |  | AGAACACCATGGGGGCACTGGAT |
|  |  |  |  | GGCGGGAATGAAGCAAGAGCCAA |
|  |  |  |  | TGACGCTCACCTTGAAAAGGTCAAT |
|  |  |  |  | GGAACCGATTCGCCAACCCATGA |
| Sequence-based reagent | *ins-3(tm3608)* genotyping primers | *C. elegans* Gene Knockout Consortium |  | GATCAGCATTGTCACCTGAC |
|  |  |  |  | TGGCAACTGATGTCCGGTAT |
| Sequence-based reagent | *ins-4(tm3620)* genotyping primers | *C. elegans* Gene Knockout Consortium |  | CCGCCCAATCCCTTTAACGT |
|  |  |  |  | GATGGCTTGTTGGACGACTG |
|  |  |  |  | TGGCGCTTGACGCATCAGTC |
|  |  |  |  | GAGTGCACTGTGTTGTGCAG |
| Sequence-based reagent | *ins-5(tm2560)* genotyping primers | *C. elegans* Gene Knockout Consortium |  | GGCTCCTTGCGCCATGATGT |
|  |  |  |  | ATCCTTGAATGCCCGTGAGT |
|  |  |  |  | GCTCCTCCCATGTTGGATTG |
|  |  |  |  | GGTTTCAGGAGGGTGACGGT |
| Sequence-based reagent | *ins-6(tm2416)* genotyping primers | *C. elegans* Gene Knockout Consortium |  | TTTACCCACCCCTTCGTGAT |
|  |  |  |  | TTGTACAAGCCACTGGGATG |
|  |  |  |  | TCGTGATGCTCCGCCTATTG |
|  |  |  |  | ACAGAGACTGATATCGGAGT |
| Sequence-based reagent | *daf-28(tm2308)* genotyping primers | *C. elegans* Gene Knockout Consortium |  | TCCGCCCACTTTGAGCTATA |
|  |  |  |  | GCACCCGATCTGACGACACT |
|  |  |  |  | GGGTTATCACTAGGAAGTTG |
|  |  |  |  | ACCGAGAGGTAGGGGTAATT |
| Sequence-based reagent | *hlh-30(tm1978)* genotyping primers | Protocol from Javier Irazoqui (University of Massachusetts Medical School) |  | TGTCACCTCGGATAGGAAGCCGG |
|  |  |  |  | TGGCGGGAAGTTCGAAAATTGTTGA |
|  |  |  |  | GCTGAAATGTTTGCTCAAAAGCGCC |
| Sequence-based reagent | *ctl-2(ok1137)* genotyping primers | Erin Cram (Northeastern University) |  | TACCCAGAAGCGTAATCCACAG |
|  |  |  |  | CATCTTGTCTGGCGAGAACTCG |
